# Activation and Induction of Antigen-Specific T Follicular Helper Cells Play a Critical Role in Live-Attenuated Influenza Vaccine-Induced Human Mucosal Anti-influenza Antibody Response

**DOI:** 10.1128/JVI.00114-18

**Published:** 2018-05-14

**Authors:** Abdullah Aljurayyan, Suttida Puksuriwong, Muhammad Ahmed, Ravi Sharma, Madhan Krishnan, Salil Sood, Katherine Davies, Devika Rajashekar, Sam Leong, Paul S. McNamara, Stephen Gordon, Qibo Zhang

**Affiliations:** a Department of Clinical Infection, Microbiology and Immunology, Institute of Infection and Global Health, University of Liverpool, Liverpool, United Kingdom; b ENT Department, Alder Hey Children's Hospital, Liverpool, United Kingdom; c ENT Department, Royal Liverpool and Broadgreen University Hospitals, Liverpool, United Kingdom; d ENT Department, Aintree University Hospital of Liverpool, Liverpool, United Kingdom; e Institute in the Park, Alder Hey Children's Hospital, Liverpool, United Kingdom; f Liverpool School of Tropical Medicine, Liverpool, United Kingdom; Icahn School of Medicine at Mount Sinai

**Keywords:** nasopharynx-associated lymphoid tissue, NALT, LAIV, T follicular helper cell, T_FH_, antibody response, influenza vaccine, mucosal immunity

## Abstract

There is increasing interest recently in developing intranasal vaccines against respiratory tract infections. The antibody response is critical for vaccine-induced protection, and T follicular helper cells (T_FH_) are considered important for mediating the antibody response. Most data supporting the role for T_FH_ in the antibody response are from animal studies, and direct evidence from humans is limited, apart from the presence of T_FH_-like cells in blood. We studied the activation and induction of T_FH_ and their role in the anti-influenza antibody response induced by a live-attenuated influenza vaccine (LAIV) in human nasopharynx-associated lymphoid tissue (NALT). T_FH_ activation in adenotonsillar tissues was analyzed by flow cytometry, and anti-hemagglutinin (anti-HA) antibodies were examined following LAIV stimulation of tonsillar mononuclear cells (MNC). Induction of antigen-specific T_FH_ by LAIV was studied by flow cytometry analysis of induced T_FH_ and CD154 expression. LAIV induced T_FH_ proliferation, which correlated with anti-HA antibody production, and T_FH_ were shown to be critical for the antibody response. Induction of T_FH_ from naive T cells by LAIV was shown in newly induced T_FH_ expressing BCL6 and CD21, followed by the detection of anti-HA antibodies. Antigen specificity of LAIV-induced T_FH_ was demonstrated by expression of the antigen-specific T cell activation marker CD154 upon challenge by H1N1 virus antigen or HA. LAIV-induced T_FH_ differentiation was inhibited by BCL6, interleukin-21 (IL-21), ICOS, and CD40 signaling blocking, and that diminished anti-HA antibody production. In conclusion, we demonstrated the induction by LAIV of antigen-specific T_FH_ in human NALT that provide critical support for the anti-influenza antibody response. Promoting antigen-specific T_FH_ in NALT by use of intranasal vaccines may provide an effective vaccination strategy against respiratory infections in humans.

**IMPORTANCE** Airway infections, such as influenza, are common in humans. Intranasal vaccination has been considered a biologically relevant and effective way of immunization against airway infection. The vaccine-induced antibody response is crucial for protection against infection. Recent data from animal studies suggest that one type of T cells, T_FH_, are important for the antibody response. However, data on whether T_FH_-mediated help for antibody production operates in humans are limited due to the lack of access to human immune tissue containing T_FH_. In this study, we demonstrate the induction of T_FH_ in human immune tissue, providing critical support for the anti-influenza antibody response, by use of an intranasal influenza vaccine. Our findings provide direct evidence that T_FH_ play a critical role in vaccine-induced immunity in humans and suggest a novel strategy for promoting such cells by use of intranasal vaccines against respiratory infections.

## INTRODUCTION

Vaccination is one of the most effective preventative measures against pathogenic infection. Despite its success, there are still many infectious diseases of humans that lack effective vaccines. New strategies to improve vaccine immunogenicity are constantly being explored. Recent studies suggested a critical role for T follicular helper cells (T_FH_) in vaccine-induced immunity (1, 2), and promoting T_FH_ has been considered a promising vaccination strategy. However, most of the current evidence supporting the importance of T_FH_ in vaccination comes from animal studies, and direct evidence from humans is limited, apart from the detection of T_FH_-like cells, which are thought to be equivalent to T_FH_, in human peripheral blood samples (3, 4). Whether this T_FH_-mediated critical help for vaccine-induced B cell antibody responses operates in humans remains largely unsubstantiated. Several recent studies reported that the presence of “T_FH_-like” cells in peripheral blood following parenteral influenza vaccination appeared to correlate with an anti-hemagglutinin (anti-HA) antibody response (5, 6).

T_FH_ are a subset of CD4^+^ T cells in secondary lymphoid tissue that provide help to cognate B cells for high-affinity antibody production in germinal centers (GC) and for long-term humoral immunity (7). T_FH_ express the chemokine receptor CXCR5 as well as the inducible costimulator (ICOS), interleukin-21 (IL-21), and the transcription factor B cell lymphoma 6 (BCL6) (8). Considering the importance of T_FH_ for B cell antibody responses, the development of novel vaccines to induce/activate T_FH_ may be an effective strategy for better vaccine efficacy in humans.

Influenza virus infects the nasopharyngeal mucosa by binding its surface HA to sialic acid receptors on the host cell (9). Intranasal vaccination has been proposed as an effective way of immunizing against influenza through induction of anti-HA antibody, which relies on the local mucosal immune tissue, i.e., nasopharynx-associated lymphoid tissue (NALT), as the induction site for immunity. Human adenoids and tonsils are major components of NALT and are known to be major induction sites for both mucosal and systemic immunity against upper respiratory tract pathogens, including influenza virus (10–13).

Live-attenuated influenza vaccines (LAIV) are administered as intranasal sprays and comprise live-attenuated influenza type A (H1N1 and H3N2) and type B viruses. LAIV have been used in a number of countries, including the United States and Canada (FluMist) (14), and in Europe (Fluenz), and have been shown to induce both mucosal and serum antibodies as well as cellular immune responses (15–17).

Although LAIV have been shown to be effective against influenza (18), limited data are available on the induction of LAIV-induced immunity in humans and on how the anti-HA antibody response is regulated by T cells. We studied the activation and induction of T_FH_ by LAIV and their role in the anti-HA antibody response in human NALT and showed that the induction of antigen-specific T_FH_ in NALT is critical for the LAIV-induced anti-influenza HA antibody response.

## RESULTS

### LAIV activates a proliferative T_FH_ response in NALT that provides critical help for anti-HA antibody production.

**FIG 1:**
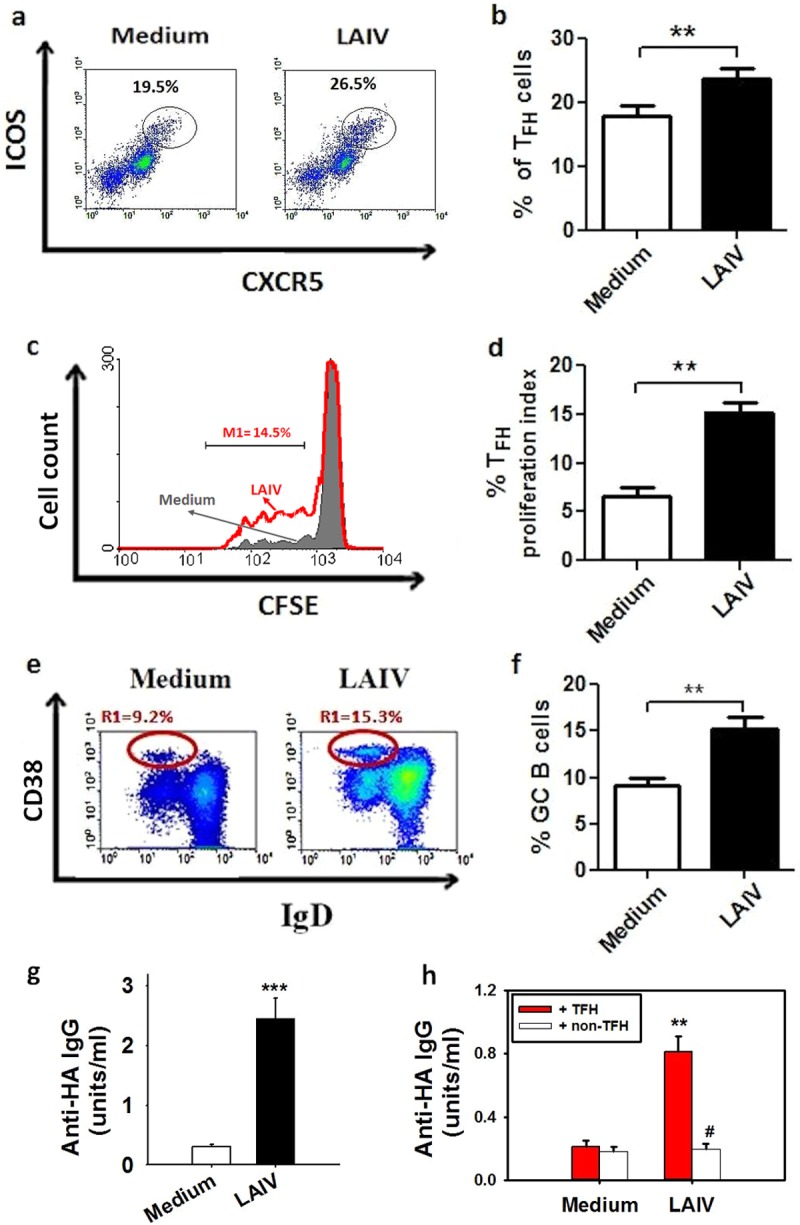
LAIV induces T_FH_ proliferation that correlates with the GC B cell response and antibody production in NALT. LAIV stimulation induced increases in T_FH_ number (a and b) and T_FH_ proliferation (c and d) in tonsillar MNC (*n* = 15 for panels b and d; **, *P* < 0.01 versus unstimulated medium controls). (a and c) Representative plots and histogram for the T_FH_ subset (CXCR5^hi^ ICOS^hi^) of CD4^+^ T cells following stimulation (day 3) (a) and for T_FH_ proliferation analyzed by CFSE staining (day 5) (red line, LAIV; gray shaded area, medium control) (c). (e and f) Increase in GC B cell number (CD19^+^ CD38^hi^ IgD^−^) in tonsillar MNC after LAIV stimulation (*n* = 13; **, *P* < 0.01 versus control). (g and h) LAIV-induced anti-HA IgG antibody production in tonsillar MNC (*n* = 20; ***, *P* < 0.01 versus control; day 8) (g) and LAIV-induced anti-HA IgG production in B cells cocultured with T_FH_ (red bars) or with non-T_FH_ cells (white bars) (*n* = 10; **, *P* < 0.01; #, *P* > 0.05 versus control) (h). Data in the bar figures are means and SE for a number of different experiments done with tonsils from different donors.

Activation of T_FH_ in NALT was examined by LAIV stimulation of adenotonsillar mononuclear cells (MNC) for 3 days, followed by enumeration of T_FH_ numbers by use of flow cytometry. As shown in Fig. 1a and b, LAIV stimulation elicited a significant increase in T_FH_ (CD4^+^ CXCR5^hi^ ICOS^hi^) number compared to that for unstimulated controls (*P* < 0.01). The T_FH_ response was further assessed by analysis of T cell proliferation by carboxyfluorescein succinimidyl ester (CFSE) cell tracing. As shown in Fig. 1c and d, stimulation of tonsillar MNC by LAIV elicited a marked T_FH_ proliferative response detected at day 5 of cell culture (*P* < 0.001). Further analysis also demonstrated a marked increase in the number of germinal center B cells (CD19^+^ CD38^+^ IgD^−^) following LAIV stimulation (*P* < 0.01) (Fig. 1e and f).

Anti-influenza antibody production was measured in the tonsillar MNC culture supernatant following LAIV stimulation for 8 days. As expected, LAIV elicited marked anti-HA antibody production (Fig. 1g), and a T cell-B cell coculture experiment demonstrated that B cells cocultured with purified T_FH_ elicited anti-HA antibody production, whereas no antibody production was shown for B cells cocultured with non-T_FH_ (CXCR5^−^ CD4^+^) cells (Fig. 1h).

### Induction of antigen-specific T_FH_ by LAIV correlates with antibody production.

**FIG 2:**
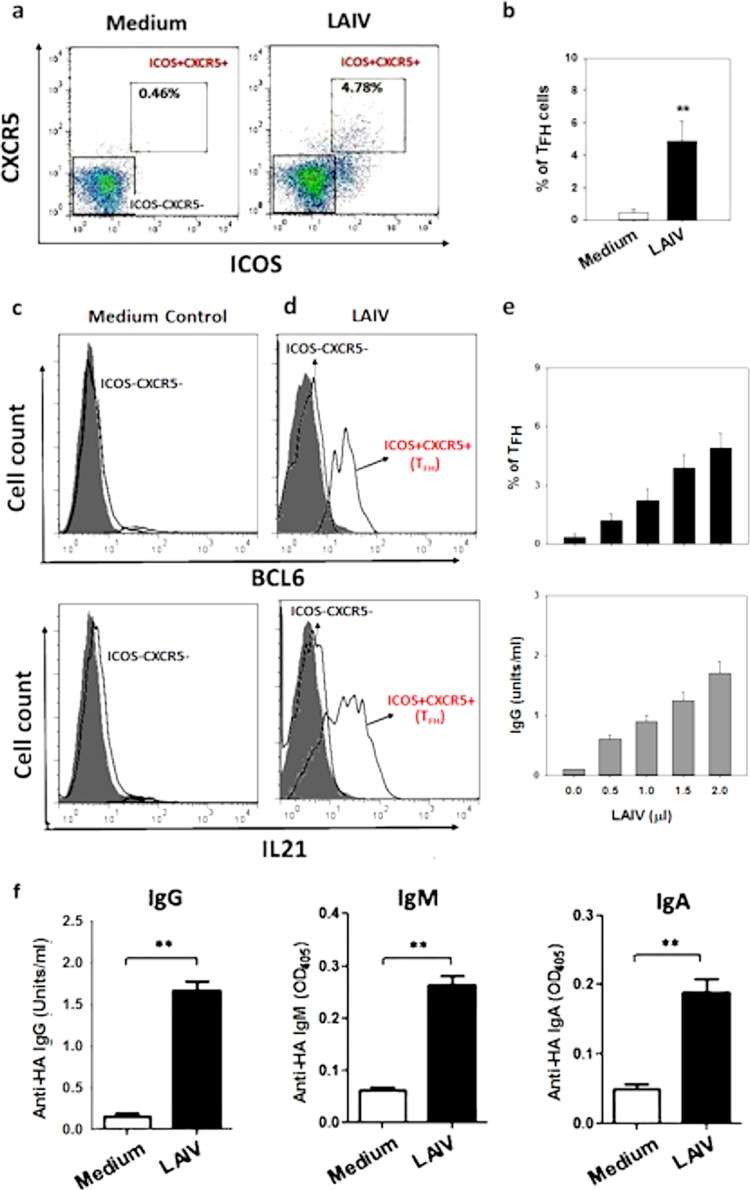
Induction of T_FH_ from naive tonsillar T cells and antibody production by LAIV. Representative plots (a) and a bar graph (b) show the induction of T_FH_ (CD4^+^ CXCR5^+^ ICOS^+^) from CD45RO^−^ MNC by LAIV compared to that for the medium control (*n* = 10; **, *P* < 0.01). (c and d) Fluorescence-activated cell sorting (FACS) histograms of BCL6 (c) and IL-21 (d) expression in LAIV-induced T_FH_ compared to that in unstimulated medium controls (isotype controls [gray shading]). (e) Dose-dependent induction of T_FH_ (day 7; top) and anti-HA IgG antibody production (day 14; bottom) from CD45RO^−^ MNC following LAIV stimulation (*n* = 6). (f) LAIV-induced anti-HA IgG, IgM, and IgA production in CD45RO^−^ MNC (day 14; *n* = 10; **, *P* < 0.01).

To determine whether LAIV induces T_FH_ differentiation from naive CD4^+^ T cells in NALT, tonsillar MNC depleted of CD45RO^+^ T cells (resulting in CD45RO^−^ MNC) were stimulated with LAIV for 7 days. The CD45RO^−^ MNC contained naive T cells but no CD45RO^+^ cells, including CXCR5^+^ T_FH_. As shown in Fig. 2a and b, LAIV stimulation of CD45RO^−^ MNC induced a marked increase in the number of CD4^+^ ICOS^+^ CXCR5^+^ cells (T_FH_) following 7 days of cell culture. The induced T_FH_ were shown to express the transcription factor BCL6 and the cytokine IL-21 (Fig. 2c and d). The induction of T_FH_ by LAIV was shown to occur in a dose-dependent fashion (Fig. 2e, top panel) and was accompanied by a dose-dependent increase in anti-HA IgG antibody production in the cell culture supernatant that was detected at day 14 (Fig. 2e, bottom panel). All 3 major antibody isotypes, including IgG, IgM, and IgA anti-HA antibodies, were detected in the culture supernatant at day 14 following LAIV stimulation (Fig. 2f).

**FIG 3:**
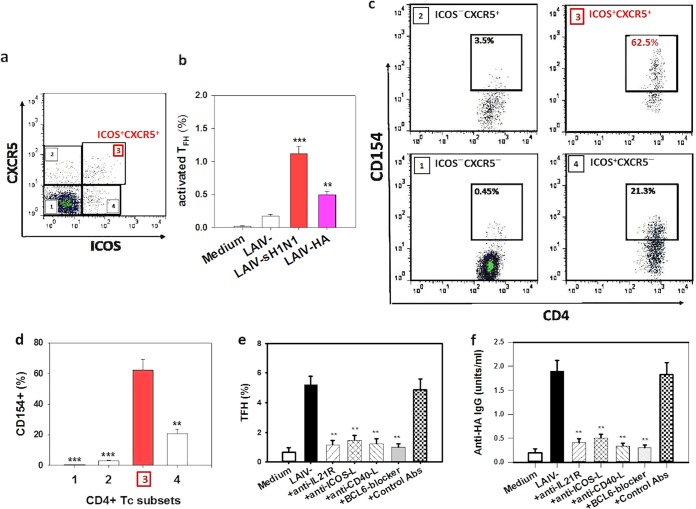
Detection of LAIV-induced antigen-specific T_FH_ and effects of IL-21, ICOS, CD40, and BCL6 signaling on T_FH_ and antibody induction. CD45RO^−^ MNC were first stimulated with LAIV for 7 days, followed by influenza virus antigen challenge with sH1N1 or HA antigen. (a) Representative plot showing activated T_FH_ (ICOS^+^ CXCR5^+^) following sH1N1 antigen challenge. (b) Representative plot showing the frequencies of activated T_FH_ (% of CD4^+^ T cells) after sH1N1 or HA challenge following prior LAIV stimulation (**, *P* < 0.01; ***, *P* < 0.001 versus LAIV stimulation alone). The medium-only negative-control level is also shown. (c and d) Representative plots (c) and frequency summary (*n* = 5) (d) for CD154 expression in the CD4^+^ T cell subsets, including T_FH_, following sH1N1 antigen challenge. (e and f) Effects of neutralizing antibodies to IL-21R, ICOS-L, and CD40-L or a BCL6 blocker on T_FH_ induction (day 7) (e) and antibody production (day 14) (f) in CD45RO^−^ MNC following LAIV stimulation (**, *P* < 0.01 versus LAIV stimulation or use of isotype control antibodies).

Having shown the induction of T_FH_ by LAIV, we next examined the specificity of the induced T_FH_ for influenza virus antigens. As CD154 is considered a reliable functional marker for antigen-activated T cells, i.e., a marker for antigen-specific T cells (5, 19–21), CD154 expression in the CD4^+^ T cell subsets was analyzed following challenge with either an inactivated seasonal H1N1 (sH1N1) virus antigen or recombinant HA. A representative dot plot demonstrating the activated T_FH_ (ICOS^+^ CXCR5^+^ cells; top right quadrant) following the antigen challenge is shown in Fig. 3a, and the frequency of activated T_FH_ (% of CD4^+^ T cells) following sH1N1 antigen or HA challenge is shown in Fig. 3b. Both antigen stimulations activated a marked increase in T_FH_ number compared to that with the nonantigen control, and as expected, the sH1N1 virus antigen challenge elicited a larger increase in T_FH_ frequency than that induced by HA (Fig. 3b). Among the activated T_FH_ following sH1N1 challenge, a large proportion (mean, 62.2%) expressed CD154 (Fig. 3c and d), demonstrating the high frequency of activated influenza virus antigen-specific T cells in these T_FH_, which was substantially higher than those of the other, non-T_FH_ CD4^+^ cell populations, i.e., 0.45% in ICOS^−^ CXCR5^−^, 3.05% in ICOS^−^ CXCR5^+^, and 20.6% in ICOS^+^ CXCR5^−^ populations (*P* < 0.001, *P* < 0.001, and *P* < 0.01, respectively) (Fig. 3c and d). Similar proportions of CD154^+^ CD4^+^ T cell populations, including CD154^+^ T_FH_, were shown following the HA antigen challenge (data not shown).

### LAIV-activated induction of T_FH_ in NALT involves IL-21, ICOS, CD40, and BCL6 signaling.

As LAIV-induced T_FH_ expressed high levels of IL-21 and ICOS, we determined whether the T_FH_ induction from naive T cells involved IL-21 receptor (IL-21R) and ICOS signaling. Coincubation of naive T cell-containing CD45RO^−^ MNC with either an IL-21R or ICOS ligand (ICOS-L) blocking antibody led to a marked reduction in T_FH_ induction by LAIV (*P* < 0.01) (Fig. 3e). Furthermore, coincubation with a CD40 ligand (CD40-L) blocking antibody or a BCL6 inhibitor also led to a marked reduction in T_FH_ induction (Fig. 3e). Finally, coincubation with anti-IL-21R, -ICOS-L, or CD40-L antibody or the BCL6 blocker inhibited the LAIV-induced anti-HA antibody production in CD45RO^−^ MNC (*P* < 0.01) (Fig. 3f).

### IL-21 production by LAIV-activated T_FH_ is critical for anti-HA antibody production.

**FIG 4:**
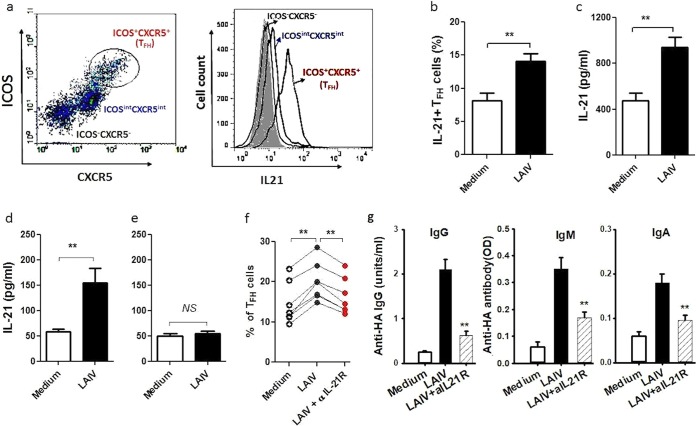
IL-21 expression in LAIV-activated T_FH_ and its effect on anti-HA antibody production. (a) Representative plots showing T_FH_ subset and IL-21 expression levels in tonsillar CD4^+^ T cells following LAIV stimulation (including isotype control data [gray shading]). (b) Increase in IL-21-producing T_FH_ (% of CD4^+^ T cells) among tonsillar MNC following LAIV stimulation (*n* = 10; **, *P* < 0.01 versus control). (c to e) IL-21 concentrations following stimulation in the culture supernatants of tonsillar MNC (*n* = 22) (c), B cells cocultured with T_FH_ (*n* = 10) (d), or non-T_FH_ cells (*n* = 10) (e) (**, *P* < 0.01 versus control; NS, not significant). (f and g) IL-21R blocking by addition of anti-IL-21R antibody to tonsillar MNC led to reductions in T_FH_ number (f) and anti-HA IgG, IgM, and IgA antibody production (g) (*n* = 8; **, *P* < 0.01).

We next examined the cellular source and production of IL-21 in tonsillar MNC following LAIV stimulation and its effects on T_FH_ activation and antibody production. Among tonsillar lymphocytes, T_FH_ were shown to be a predominant source of IL-21 (Fig. 4a). Following LAIV stimulation, there was an increase of IL-21-producing T_FH_ in tonsillar MNC (Fig. 4b), together with a marked increase in the IL-21 concentration in the MNC culture supernatant (Fig. 4c). Further, the increase in IL-21 concentration was shown for the coculture of T_FH_ and B cells (Fig. 4d) but was not seen for the coculture of non-T_FH_ cells with B cells following LAIV stimulation (Fig. 4e).

IL-21 receptor blocking using an anti-IL-21R antibody abrogated the increase of T_FH_ number in tonsillar MNC elicited by LAIV stimulation (Fig. 4f), followed by a significant reduction in anti-HA antibody production in tonsillar MNC (Fig. 4g).

### Activation of T_FH_-like cells in PBMC by LAIV.

**FIG 5:**
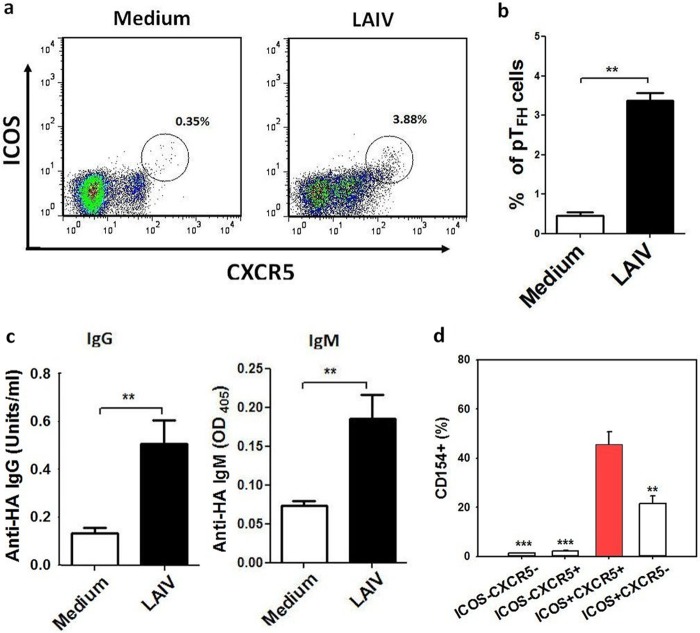
Activation of T_FH_-like cells in PBMC. (a) Representative plots show the increase of T_FH_-like cells (CD4^+^ CXCR5^+^ ICOS^+^) in PBMC following stimulation by LAIV for 3 days compared to their level in the medium control. (b) LAIV-induced increase in T_FH_-like cells in PBMC compared to the control level (*n* = 10; **, *P* < 0.01). (c) Anti-HA IgG and IgM antibody production in PBMC culture supernatant following LAIV stimulation (*n* = 10; **, *P* < 0.01). (d) Frequency of antigen-specific CD154^+^ T_FH_-like cells (% of CD4^+^ T cells; red bar) in PBMC following LAIV stimulation and subsequent sH1N1 antigen challenge compared to the frequencies of other CD4^+^ T cell subpopulations, as indicated (*n* = 4; **, *P* < 0.01; ***, *P* < 0.001).

Recent studies suggested that there are T_FH_-like cells in peripheral blood that express CXCR5 and ICOS and have similar B cell helper functions (4, 5, 22–24). To determine whether LAIV activates T_FH_-like cells and antibody production in peripheral blood, freshly isolated peripheral blood mononuclear cells (PBMC) were stimulated with LAIV for up to 14 days, followed by flow cytometry and antibody detection. As shown in Fig. 5a and b, LAIV stimulation induced an increase of T_FH_-like (CXCR5^+^ ICOS^+^) CD4^+^ T cells in PBMC (at day 7), followed by the detection of anti-HA IgG and IgM antibodies in the PBMC culture supernatants (Fig. 5c). The activation of influenza virus antigen-specific T_FH_-like cells by LAIV was demonstrated by the finding that a major proportion (mean, 45.6%) of these cells expressed CD154 following the H1N1 antigen challenge, and this proportion was markedly higher than those of the other, non-T_FH_ cell populations (Fig. 5d).

## DISCUSSION

LAIV is thought to replicate in the upper respiratory tract to induce immunity through the local immune tissue NALT, and it was shown to replicate in nasal epithelial cells (25). As part of the mucosal immune system in the human nasopharynx, adenotonsillar tissue has a surface reticular epithelial cell layer in which epithelial cells are mixed with other cells, including a large number of B cells. Many B cells infiltrating the epithelial layer exhibit memory B cell markers and have great antigen-presenting potential (26, 27). In our adenotonsillar MNC culture, the predominant cell populations were lymphocytes, over 50% of which were B cells (28). We previously showed that a modified vaccinia virus Ankara (MVA)-vectored influenza vaccine predominantly infected tonsillar B cells, which were also the major cells presenting vaccine antigens (29). It is likely that tonsillar B cells are a major cell population involved in LAIV replication and antigen presentation to T cells and that this B and T cell interaction contributes to the vaccine-induced response in NALT. Our recent pilot data showed a time-dependent increase in HA expression in tonsillar B cells following LAIV stimulation, consistent with virus replication in tonsillar B cells. Fetal bovine serum (FBS; 10%) was used in our cell culture, and we did not find any evidence suggesting a blockade of LAIV replication (data not shown).

In this study, we demonstrated the activation and induction of antigen-specific T_FH_ in human nasopharynx immune tissue by LAIV and showed that T_FH_ are critical for the LAIV-induced B cell anti-HA antibody response in the immune induction tissue of children and adults.

We showed a marked increase in the T_FH_ number in tonsillar MNC following stimulation by LAIV (Fig. 1a and b). With CFSE cell tracing, we also demonstrated T_FH_ proliferation following stimulation (Fig. 1c and d). The increase in T_FH_ number was accompanied by the production of anti-HA antibodies in tonsillar MNC (Fig. 1g). We further demonstrated in the cell coculture experiment that purified T_FH_ from tonsillar MNC helped B cell anti-HA antibody production, whereas non-T_FH_ cells did not (Fig. 1h). These results support the hypothesis that T_FH_ provide critical help for LAIV-induced B cell anti-HA antibody production in human NALT.

Together with the increases in T_FH_ number and antibody production following LAIV stimulation, a marked increase in GC B cells was also seen in tonsillar MNC (Fig. 1e and f). This is consistent with the assumption that LAIV activates T_FH_ which support GC B cell proliferation and differentiation for antibody production. It was reported previously that the number of T_FH_ correlated with that of GC B cells in NALT (20). These data are concordant with previous reports on mouse models showing that GC B cells correlated with the appearance of T_FH_ after influenza virus infection (30) and that the magnitude of the T_FH_ response was directly correlated with the GC B cell response (31, 32).

We next examined the induction of influenza virus antigen-specific T_FH_ from naive T cells by LAIV by using T_FH_-depleted CD45RO^−^ MNC. Seven days following LAIV stimulation, we observed a dose-dependent increase in the number of newly differentiated T_FH_ (CXCR5^+^ ICOS^+^) that coexpressed BCL6 and IL-21, which was followed by the detection of anti-HA antibody at day 14 (Fig. 2a to e). Both BCL6 and IL-21 are known to be essential for T_FH_ differentiation from naive T cells in animal studies (8, 33, 34). Our results support the hypothesis that T_FH_ induction in human immune tissue also requires BCL6 and IL-21. Indeed, further experiments with a BCL6 blocker and an IL-21 blocking antibody demonstrated marked reductions of T_FH_ induction from naive tonsillar T cells, confirming a critical role for BCL6 and IL-21 in T_FH_ induction. We also showed that ICOS signaling blocking inhibited ICOS activation and T_FH_ induction, supporting the hypothesis that ICOS activation is required for T_FH_ differentiation. It has been suggested that CD4^+^ T cells utilize ICOS–ICOS-L interactions to upregulate IL-21 production to contribute to T_FH_ induction (34). Our finding that the CD40-L blocking antibody abrogated T_FH_ induction is in line with the hypothesis that B and T cell cognate interaction through CD40–CD40-L signaling is critical for T_FH_ induction.

One finding we observed was that CD45RO^+^ cell depletion, which removes memory T cells from tonsillar MNC, markedly reduced anti-HA antibody production analyzed at day 8 (memory response). The fact that anti-HA IgG in whole tonsillar MNC following vaccine stimulation could readily be detected at a high level at day 8 (Fig. 1g), whereas in memory T cell-depleted MNC the antibody production could be detected only at around day 14, and at a lower level (Fig. 2f), suggests the presence of influenza virus-specific memory T/B cells in tonsillar MNC. In this study, although tonsillar tissues were from nonvaccinated donors, it is likely that some of the donors had experienced an influenza virus infection previously and had influenza virus-specific memory T/B cells. Therefore, the presence of memory T cells, including T_FH_, in tonsillar MNC helped the memory B cell response following LAIV stimulation.

Further to the reduction of T_FH_ induction following BCL6, IL-21, ICOS, and CD40 signaling blocking, we showed that the blockade of these signals led to diminished anti-HA antibody production, supporting the critical involvement of these pathways in T_FH_ induction and T_FH_-mediated B cell antibody production. The induction of influenza virus antigen-specific T_FH_ by LAIV was demonstrated by the detection of the antigen-specific CD4^+^ T cell activation marker CD154, which was expressed in a large proportion of the induced T_FH_ following influenza virus antigen challenge (Fig. 3). This finding is consistent with a report by Bentebibel et al. demonstrating an increase in influenza virus antigen-specific T_FH_-like cells in peripheral blood following an inactivated vaccine immunization in humans (5).

Studies with an animal model demonstrated a critical role of IL-21 in T_FH_ activation, and T_FH_ were also shown to be the predominant source of IL-21 (33, 35). We showed here that stimulation of tonsillar MNC with LAIV activated marked increases in IL-21-producing T_FH_ and in the IL-21 concentration in the cell culture supernatant. These results are consistent with the assumption that T_FH_ are a major cellular source of IL-21 in human tonsillar lymphocytes, as we found no significant IL-21 production in the absence of T_FH_ in the T cell-B cell coculture experiment (Fig. 4). We also demonstrated that newly differentiated T_FH_ following LAIV stimulation expressed a high level of IL-21 (Fig. 2). As tonsillar T_FH_ are also known to express IL-21R (34), this coexpression of IL-21 and IL-21R by tonsillar T_FH_ supports the hypothesis that IL-21 acts in an autocrine loop fashion in the vaccine-induced T_FH_ differentiation and function in human NALT. Indeed, we showed that blocking IL-21 signaling by use of an IL-21R neutralizing antibody inhibited both activation and differentiation of T_FH_ induced by LAIV, which diminished anti-HA antibody production. Thus, our results provide direct supporting evidence that IL-21 is crucial for vaccine-induced T_FH_ differentiation and T_FH_-dependent B cell antibody production in human immune tissue.

Consistent with recent reports that there is an increase in T_FH_-like cells in human peripheral blood following parenteral influenza vaccination which correlates with the anti-HA antibody response (5, 6), we showed that LAIV stimulation of PBMC also induced an increase in CXCR5^+^ T_FH_-like cells together with the production of anti-HA antibodies in the PBMC (Fig. 5). The activation of influenza virus antigen-specific T_FH_ in PBMC by LAIV was demonstrated by the expression of the antigen-specific T cell activation marker CD154 upon antigen challenge. These results support the concept that there are T_FH_-like cells in peripheral circulation which are functionally similar to T_FH_ found in lymphoid tissue, such as NALT, and provide help to B cells for antibody production in an IL-21- and ICOS-dependent manner (4).

In conclusion, we have demonstrated the induction of antigen-specific T_FH_ in human immune tissue by use of an intranasal influenza vaccine and have shown their critical role in the anti-influenza virus HA antibody response. Our results suggest that promoting antigen-specific T_FH_ in human NALT by use of intranasal vaccines may provide an effective vaccination strategy against respiratory infections in humans.

## MATERIALS AND METHODS

### Patients and samples.

Patients (2 to 30 years of age) undergoing adenoidectomy and/or tonsillectomy due to upper airway obstruction were recruited, and adenotonsillar tissues were obtained following operation. A peripheral blood sample was also obtained before operation. The tonsillar tissues were placed in Hanks' balanced salt solution (HBSS) and transported to the laboratory. Tissue samples exhibiting any signs of gross inflammation were excluded. Patients with any known immunodeficiency were excluded from the study. Subjects who previously received influenza vaccination were also excluded from the study. The Liverpool Pediatric Research Ethics Committee approved the study (approval 08/H1002/92), and written informed consent was obtained in each case.

### LAIV vaccine and influenza virus antigens.

An intranasal LAIV (FluMist, 2009–2010) that included A/Brisbane/59/2007 (H1N1), A/Brisbane/10/2007 (H3N2), and influenza B virus strains was obtained from BEI Resources (ATCC, Manassas, VA). A 0.2-ml aliquot of the LAIV contains approximately 10^7^ fluorescent-focus units (FFU) of each strain, and we used 2 μl/ml (∼10^5^ FFU/ml) for cell stimulation, which was a predetermined optimal concentration for the activation of the anti-HA antibody response following dose titration experiments. Antigen from a seasonal A/Brisbane/59/2007 H1N1 influenza virus (sH1N1), which was inactivated by use of β-propiolactone and partially purified (36), was obtained from the National Institute for Biological Standards and Control (NIBSC), United Kingdom. This inactivated sH1N1 antigen contained 83 μg/ml of HA. Purified recombinant HA of sH1N1 (ATCC) was used for HA antigen stimulation, in addition to the coating antigen, for anti-HA antibody measurement by enzyme-linked immunosorbent assay (ELISA). The recombinant HA protein contained a C-terminal histidine tag, was produced in High Five insect cells by use of a baculovirus expression vector system and purified from cell culture supernatant by immobilized-metal affinity chromatography (IMAC), and contained a trimerizing (foldon) domain (37).

### Cell culture and stimulation.

Mononuclear cells (MNC) from adenotonsillar tissues were isolated using Ficoll density centrifugation (38, 39). In some experiments, tonsillar MNC were depleted of effector and memory (CD45RO^+^) T cells by use of CD45RO microbeads and magnetic cell sorting (Miltenyi) by passing cells through the depletion column twice as described previously (40, 41). The depletion of CD45RO^+^ cells from tonsillar MNC removed T_FH_ (>98%). Unfractionated whole MNC or CD45RO^+^ cell-depleted MNC (4 × 10^6^/ml) were cultured in RPMI 1640 medium supplemented with 10% fetal bovine serum (FBS), streptomycin (50 μg/ml), and penicillin (50 U/ml) (Sigma) in the presence of LAIV (2 μl/ml unless otherwise stated) for up to 14 days. Cells were collected at predefined time points for analysis of T_FH_ by flow cytometry. Cell culture supernatants were collected for measurement of cytokine and antibody production by ELISA.

### Flow cytometry analysis of T_FH_, cell proliferation, and intracellular cytokine expression.

For T_FH_ identification, tonsillar MNC were stained with anti-human CD3, CD4, CXCR5, and ICOS antibodies, followed by flow cytometry, and CD4^+^ CXCR5^hi^ ICOS^hi^ cells were identified as T_FH_ (42, 43). The tonsillar lymphocytes, gated for analysis based on typical FSC/SSC characteristics and singlet selection, typically had a viability of >98% as examined by propidium iodide staining. Expression of BCL6, a master transcription factor for T_FH_ differentiation (8), in newly induced T_FH_ was analyzed by intracellular staining with an anti-human BCL6 antibody after cell fixation/permeabilization following the manufacturer's instructions (eBioscience). Cell proliferation was examined by carboxyfluorescein succinimidyl ester (CFSE) staining of tonsillar MNC (Molecular Probes), followed by cell stimulation for 5 days and by flow cytometry (40, 41). Briefly, tonsillar MNC were labeled with CFSE (at 37°C for 8 min) and resuspended in RPMI medium before cell stimulation with LAIV (2 μl/ml) for 5 days. T_FH_ proliferation was then examined by analysis of CFSE dilution in T_FH_ (CXCR5^hi^ ICOS^hi^ cells) by flow cytometry. Intracellular cytokine expression, e.g., IL-21 expression, was analyzed following a standard intracellular staining procedure including cell permeabilization as described previously (39). Flow cytometry data were analyzed using FlowJo software.

The germinal center (GC) B cell subset was analyzed by flow cytometry with a combination of fluorescence-labeled anti-human CD19, CD38, and IgD antibodies and identified as CD19^+^ CD38^hi^ IgD^−^ cells.

### Analysis of antigen-specific T_FH_ induction.

T_FH_ differentiation/induction from naive tonsillar T cells by LAIV was examined using CD45RO^+^ cell-depleted MNC (which resulted in CD45RO^−^ MNC) as described earlier. The CD45RO^−^ MNC (with T_FH_ removed but naive T cells retained) were stimulated with LAIV (2 μl/ml unless stated otherwise) and cultured for 7 days before flow cytometric analysis of T_FH_, including CXCR5, ICOS, and BCL6 expression levels. For the detection of induced influenza virus antigen-specific T_FH_ after LAIV stimulation, the cells (at day 7) were washed and incubated for 24 h in fresh culture medium only, followed by antigen challenge with sH1N1 virus antigen or recombinant HA (1 μg/ml) for 6 h in the presence of brefeldin A. The cells were then stained for T_FH_, including CD4, CXCR5, ICOS, and intracellular CD154 expression, after cell fixation/permeabilization, with detection of antigen-specific T cells by flow cytometry (19–21).

To determine if IL-21, ICOS, CD40, and BCL6 signaling pathways are involved in the activation/induction of T_FH_, neutralizing/blocking antibody to IL-21R, ICOS-L, or CD40-L or a BCL6 inhibitor was coincubated with tonsillar MNC before LAIV stimulation**.** Briefly, recombinant human IL-21R–Fc chimera, anti-ICOS-L (R&D Systems), or anti-CD40-L (InvivoGen) antibody, an isotype control (10 μg/ml), or a BCL6 inhibitor (79-6; Calbiochem) (50 μM) was coincubated with tonsillar MNC or CD45RO^−^ MNC for 1 h prior to stimulation by LAIV. The BCL6 inhibitor 79-6 is a cell-permeative compound that selectively inhibits the transcriptional repression activity of BCL6. The MNC were then cultured for up to 7 to 14 days before analysis for T_FH_ and anti-HA antibody production.

### Measurement of HA-specific antibodies.

Production of anti-HA IgG, IgM, and IgA antibodies to sH1N1 virus in cell culture supernatants was measured as previously described (44, 45). In brief, ELISA plates were coated with recombinant sH1N1 HA overnight. Following blocking, cell culture supernatants were added and incubated for 2 h. Alkaline phosphatase-conjugated anti-human IgG, IgM, or IgA antibody was then added and incubated. Following the addition of pNPP substrate, color development was read by determining the optical density at 405 nm (OD_405_) at 1 h, and data were analyzed using DeltaSoft software. Intravenous immunoglobulin (IVIG; Intratect) containing high titers of anti-sH1N1 HA IgG antibody was used as a reference standard for IgG antibody. Anti-HA IgM and IgA antibody titers were expressed as OD values (read at 30 min), as no reference standard was available.

### Cell purification and T_FH_-B cell coculture.

Tonsillar T_FH_ and B cells were purified by magnetic cell sorting as described previously (42). Briefly, B cells were purified by negative selection using a B cell purification kit (EasySep; Stemcell), which yielded a B cell purity of >99%. For T_FH_ purification, CD4^+^ T cells were first isolated by negative selection, followed by positive selection of CXCR5^hi^ (T_FH_) by use of a biotin anti-CXCR5 antibody. The amount of anti-CXCR5 antibody was optimized to ensure that only CXCR5^hi^-expressing cells were selected (purity of >95%). CXCR5^−^ CD4^+^ T (non-T_FH_) cells were isolated from CD4^+^ T cells by negative selection using an optimized amount of anti-CXCR5 antibody (purity of >99%). Purified B cells were cocultured (1:1 ratio) with either purified T_FH_ or non-T_FH_ cells at 5 × 10^5^ cells/ml in the presence of LAIV. The cells were cultured for 10 days, and cell culture supernatants were collected for anti-HA antibody analysis.

### Statistical analysis.

Means and standard errors (SE) are given unless indicated otherwise. Differences between two groups were analyzed using Student's *t* test, and the paired *t* test was used for paired samples. Statistical analysis was performed using GraphPad Prism 5 software. *P* values of <0.05 were considered statistically significant.
